# Robotic low anterior resection for rectal cancer with side-to-end anastomosis in a patient with anal stenosis

**DOI:** 10.1186/s12957-021-02121-9

**Published:** 2021-01-13

**Authors:** Yosuke Tajima, Tsunekazu Hanai, Hidetoshi Katsuno, Koji Masumori, Yoshikazu Koide, Keigo Ashida, Hiroshi Matsuoka, Junichiro Hiro, Tomoyoshi Endo, Tadahiro Kamiya, Yongchol Chong, Kotaro Maeda, Ichiro Uyama

**Affiliations:** 1 Department of Gastrointestinal Surgery, Fujita Health University, 1-98 Dengakugakubo, Kutsukake-cho, Toyoake, Aichi 470-1192 Japan; 2 International Medical Center, Fujita Health University Hospital, 1-98 Dengakugakubo, Kutsukake-cho, Toyoake, Aichi 470-1192 Japan

**Keywords:** Side-to-end anastomosis, Rectal cancer, Anal stenosis, Robotic surgery, Low anterior resection

## Abstract

**Background:**

Colorectal anastomosis using the double stapling technique (DST) has become a standard procedure. However, DST is difficult to perform in patients with anal stenosis because a circular stapler cannot be inserted into the rectum through the anus. Thus, an alternative procedure is required for colorectal anastomosis.

**Case presentation:**

A 78-year-old woman presented with bloody stool. Colonoscopy and computed tomography revealed advanced low rectal cancer without lymph node or distant metastasis. We initially planned to perform low anterior resection using a double stapling technique or transanal hand-sewn anastomosis, but this would have been too difficult due to anal stenosis and fibrosis caused by a Milligan-Morgan hemorrhoidectomy performed 20 years earlier. The patient had never experienced defecation problems and declined a stoma. Therefore, we inserted an anvil into the rectal stump and fixed it robotically with a purse-string suture followed by insertion of the shaft of the circular stapler from the sigmoidal side. In this way, side-to-end anastomosis was accomplished laparoscopically. The distance from the anus to the anastomosis was 5 cm. The patient was discharged with no anastomotic leakage. Robotic assistance proved extremely useful for low anterior resection with side-to-end anastomosis.

**Conclusion:**

Performing side-to-end anastomosis with robotic assistance was extremely useful in this patient with rectal cancer and anal stenosis.

## Background

Colorectal anastomosis using the double stapling technique (DST) has become a standard procedure because it is technically easy to perform and has low risk of contamination. However, DST is difficult to perform in patients with benign anal stenosis because a circular stapler cannot be inserted into the rectum through the anus. In this situation, an alternative procedure is required for performing colorectal anastomosis. Here, we report the case of a patient with rectal cancer and anal stenosis in whom robot-assisted side-to-end anastomosis was successfully performed.

## Case presentation

The patient was a 78-year-old woman who presented with bloody stool. Colonoscopy and computed tomography revealed T3 low rectal cancer without lymph node or distant metastasis. Low anterior resection with conventional DST or a transanal hand-sewn anastomosis was initially planned but would have been difficult to perform because of anal stenosis and fibrosis caused by a Milligan-Morgan hemorrhoidectomy performed 20 years earlier. The patient had never experienced any defecation problems and declined a stoma. Therefore, we planned to insert an anvil into the rectal stump intracorporeally and perform side-to-end anastomosis. However, fixing the anvil using laparoscopic instruments seemed to be difficult, so we used a purse-string suture to fix the anvil with the aid of a da Vinci Xi surgical system (Intuitive Surgical Inc., Sunnyvale, CA, USA). We did not choose neoadjuvant radiotherapy to avoid exacerbation of anal stenosis due to radiation.

**Fig. 1:**
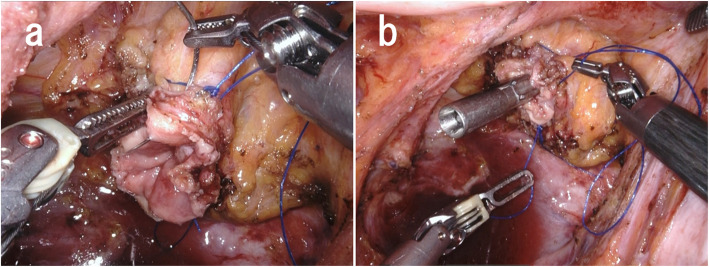
**a** Robot-assisted placement of a purse-string suture that was hand-sewn using 2-0 PROLENE. **b** The anvil was inserted into the rectal stump robotically and fixed

**Fig. 2:**
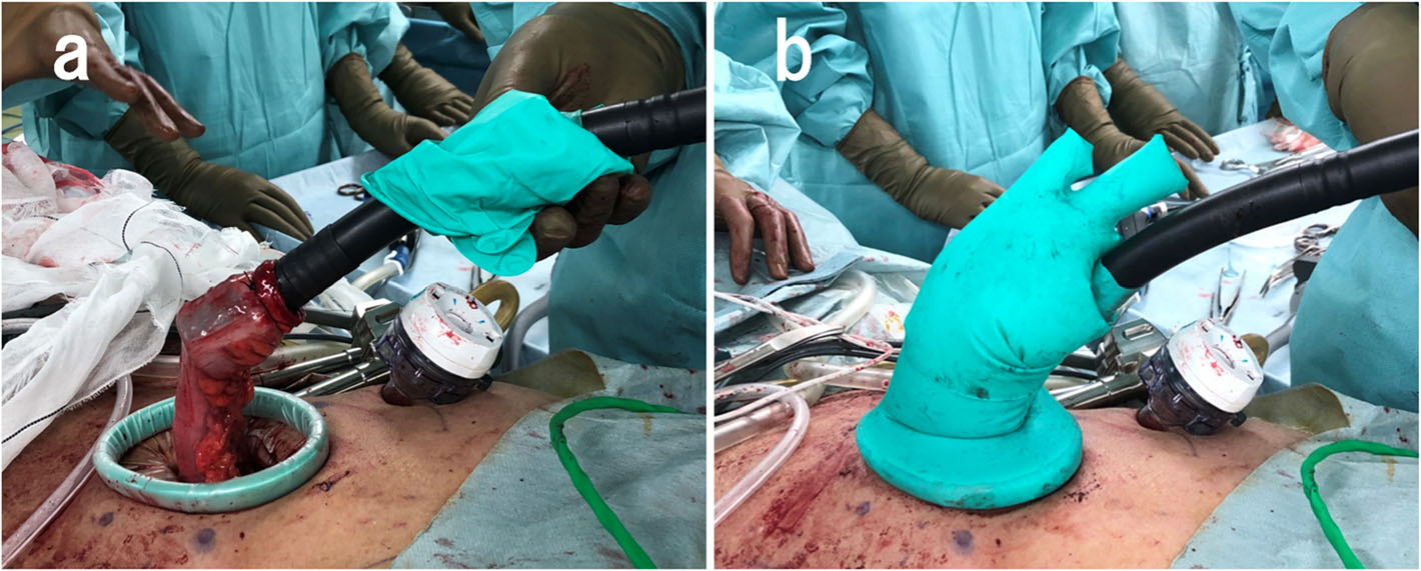
**a** The shaft of the circular stapler was introduced through the sigmoidal stump and inserted via the umbilical wound. **b** A surgical glove was attached to the wound retractor and the shaft of the circular stapler to maintain pneumoperitoneum

**Fig. 3:**
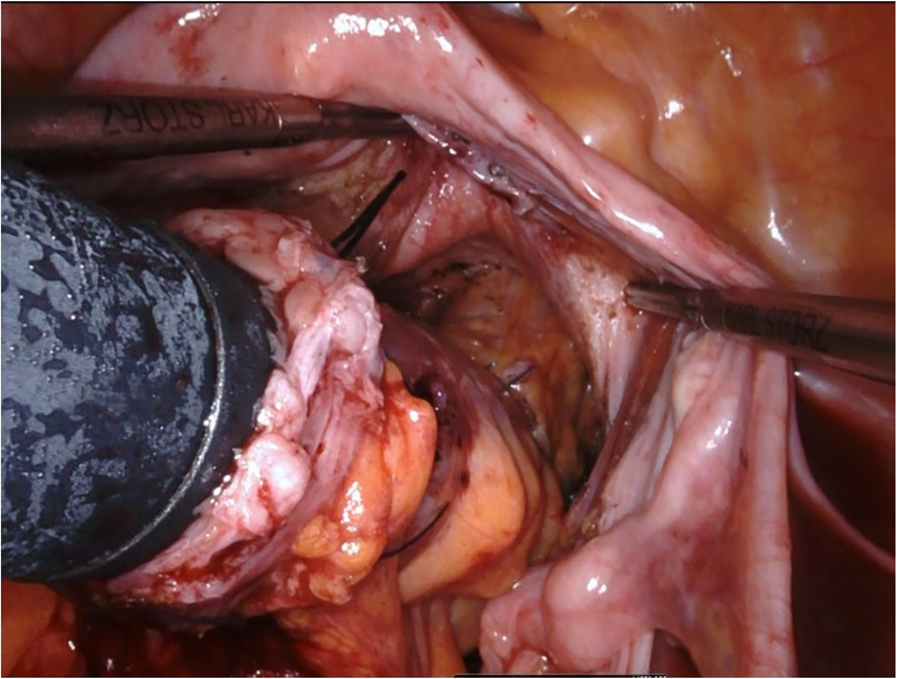
Side-to-end anastomosis was performed intracorporeally

**Fig. 4:**
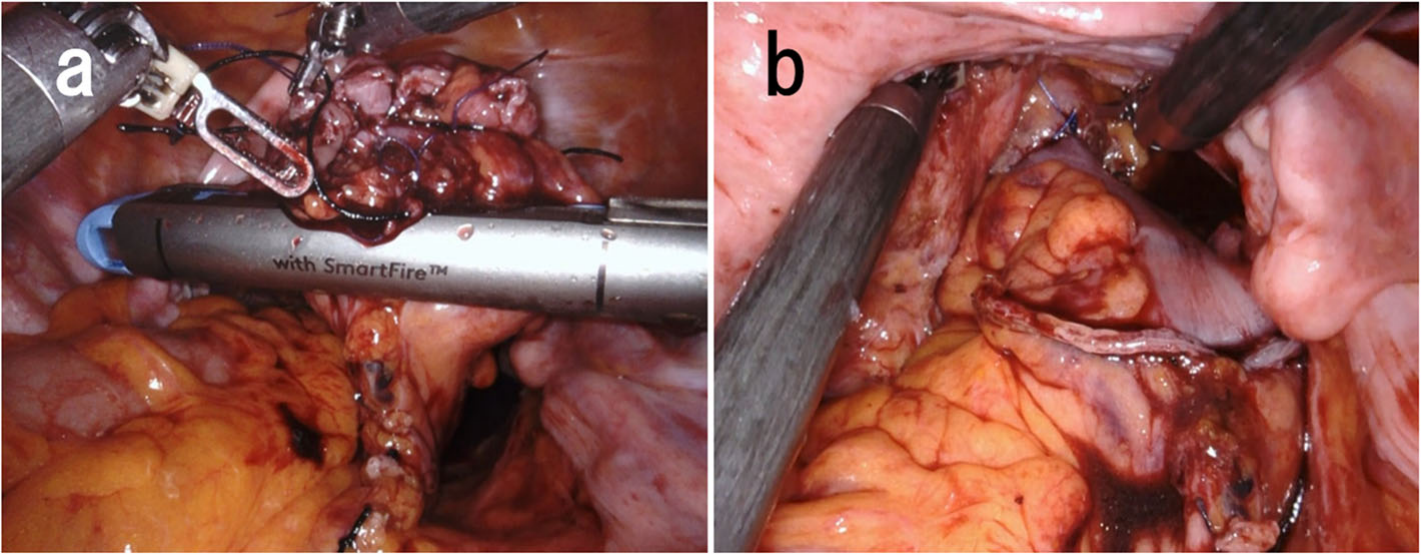
**a**, **b** The sigmoidal stump was resected using a robotic linear stapler to construct a 3-cm blind end

Robotic low anterior resection was performed as in a previous report [1]. The patient was placed right side down in the Trendelenburg position. One 12-mm camera port, three 8-mm robotic ports, and one laparoscopic assistant port were placed. Medial-to-lateral dissection of the sigmoidal mesocolon with high ligation of the inferior mesenteric artery was performed, and the sigmoid colon was separated from the lateral attachment. Total mesorectal excision with a nerve-preserving technique was achieved. After clamping of the rectum distal to the tumor for irrigation with 2.0 L of saline to prevent implantation of exfoliated cancer cells (ECCs), the lower rectum was transected more than 3 cm distal to the tumor using a robotic linear stapler (SureForm 60 Blue, Intuitive Surgical Inc.). After the staple line of the rectal stump was resected, a purse-string suture was hand-sewn robotically using 2-0 PROLENE. The anvil was then inserted into the rectal stump and fixed with robotic assistance (Fig. 1). Robotic assistance was very helpful for suturing in the narrow pelvis. Next, more than 10 cm of the sigmoid colon proximal to the tumor was transected extracorporeally. The shaft of the circular stapler (ECS25A; Ethicon, Somerville, NJ, USA) was introduced through the sigmoidal stump and inserted via the umbilical wound. A surgical glove was attached to the wound retractor and the shaft of the circular stapler to maintain pneumoperitoneum (Fig. 2). The camera was inserted via another port, after which a side-to-end anastomosis was performed laparoscopically (Fig. 3). The sigmoidal stump was resected robotically using a linear stapler to construct a 3-cm blind end (Fig. 4). The distance from the anus to the anastomosis was 5 cm. Intraoperative air leak test was negative, and a Penrose drain was inserted into the anus for the prevention of anastomotic leakage. The operating time was 459 min, and the blood loss was 12 mL. Pathological examination revealed pT3, pN0, cM0, and pStage IIA disease and confirmed an R0 resection. The patient was discharged from the hospital with no anastomotic leakage or other complications. Neither recurrence nor problems with defecation including “low anterior resection syndrome” have been noted in the 5 months since surgery.

## Discussion

Colorectal anastomosis with DST is a widely used procedure with several advantages, including its technical ease and low risk of contamination [2–4]. However, DST also has a few drawbacks, one of which is that insertion of a circular stapler via the anus is difficult in the presence of anal stenosis. For example, Miller et al. found that a circular stapler with a 31-mm cartridge could not be passed through the anus in 11.5% of low anterior resection procedures [5]. Our patient had severe anal stenosis and fibrosis as the result of a previous Milligan-Morgan hemorrhoidectomy but had no defecation problems and declined a stoma. In view of the risks of anal injury due to insertion of instrumentation and anal dysfunction because of excessive anal dilatation, another type of anastomosis was required.

Various anastomotic methods other than DST have been used after low anterior resection, including transanal hand-sewn anastomosis, functional end-to-end anastomosis, side-to-side anastomosis, colonic J-pouch anastomosis, coloplasty, and side-to-end anastomosis [6]. In our case, we considered that a transanal hand-sewn anastomosis would be too technically challenging because of the anal stenosis and that a functional end-to-end or side-to-side anastomosis would be difficult because of the shortness of the rectal stump. However, we believed that a colonic J-pouch anastomosis, coloplasty, or side-to-end anastomosis could solve these problems by allowing us to fix the anvil to the rectal stump intracorporeally and to insert the circular stapler through the proximal stump [6] or vertical incision without the need for an extra incision in the colon.

It is still unclear whether a J-pouch anastomosis, coloplasty, or side-to-end anastomosis is the optimal procedure in this situation. All these procedures achieve better anal function than end-to-end anastomosis with DST [7, 8]. However, anastomotic leakage occurs significantly more often in coloplasty than in J-pouch anastomosis [8]. Moreover, J-pouch or side-to-end anastomosis results in less anastomotic leakage because of better blood flow to the anastomotic site [9]. A J-pouch anastomosis is technically and anatomically more challenging to perform because it requires a longer segment of colon, a wide pelvis, and an extended operating time [10]. Therefore, we opted for a side-to-end anastomosis in this case. We chose a 3-cm limb because of the finding in a randomized trial that a shorter limb had a better functional outcome than a longer limb [11]. This procedure may have prevented anastomotic leakage and defecation problems in this case.

In our case, the anvil was fixed to the rectal stump using a purse-string suture that was hand-sewn robotically. This procedure is technically more demanding when performed in conventional laparoscopic surgery and is more invasive when performed in open surgery. Robotic surgery has overcome the limitations of laparoscopic and open rectal surgery by providing stable camerawork, three-dimensional magnified views, and articulating instruments while canceling out tremor. We have been performing robot-assisted colorectal surgery for more than 10 years with favorable short- and long-term outcomes [1]. Therefore, we had sufficient experience and knowledge to be able to perform successful robotic suturing and tying deep in the narrow pelvis in this case.

Intracorporeal opening of a rectal stump for fixing of the anvil head has the risk of spilling ECCs into the rectal lumen. In a report by Maeda et al., ECCs were detected in the first-washout samples in 29 of 30 patients with rectal cancer [12]. However, in another study, no ECCs were detected after washout with 1.5 L of saline in patients with a tumor located below the peritoneal reflection, and only a small number were detected after washout with 2.0 L of saline in patients with a tumor located above the peritoneal reflection [12]. Furthermore, Kodeda et al. showed that rectal washout significantly decreased the risk of local recurrence [13]. Therefore, we performed rectal washout with 2.0 L of saline before opening the rectal stump to prevent peritoneal dissemination or anastomotic recurrence.

## Conclusion

Performing side-to-end anastomosis with robotic assistance was extremely useful in this patient with rectal cancer and anal stenosis.

## Data Availability

The datasets obtained and/or analyzed in this study are available from the corresponding author on reasonable request.

## Abbreviations

DST: Double stapling technique
ECCs: Exfoliated cancer cells
